# Highly Efficient and Stable Blue Organic Light‐Emitting Diodes based on Thermally Activated Delayed Fluorophor with Donor‐Void‐Acceptor Motif

**DOI:** 10.1002/advs.202106018

**Published:** 2022-02-27

**Authors:** Dongdong Zhang, Yoshimasa Wada, Qi Wang, Hengyi Dai, Tianjiao Fan, Guoyun Meng, Jinbei Wei, Yuewei Zhang, Katsuaki Suzuki, Guomeng Li, Lian Duan, Hironori Kaji

**Affiliations:** ^1^ Key Lab of Organic Optoelectronics and Molecular Engineering of Ministry of Education Department of Chemistry Tsinghua University Beijing 100084 P. R. China; ^2^ Institute for Chemical Research Kyoto University Uji Kyoto 611‐0011 Japan; ^3^ Beijing National Laboratory for Molecular Sciences CAS Research/Education Center for Excellence in Molecular Sciences Institute of Chemistry Chinese Academy of Sciences Beijing 100190 P. R. China

**Keywords:** 9*H*‐xanthen‐9‐one, blue‐shifted emission, donor‐void‐acceptor, high efficiency, thermally activated delayed fluorescence

## Abstract

Thermally activated delayed fluorophores (TADF) with donor–acceptor (D‐A) structures always face strong conjugation between donor and acceptor segments, rendering delocalized new molecular orbitals that go against blue emission. Developing TADF emitters with blue colors, high efficiencies, and long lifetimes simultaneously is therefore challenging. Here, a D‐void‐A structure with D and A moieties connected at the void‐position where the frontier orbital from donor and acceptor cannot be distributed, resulting in nonoverlap of the orbitals is proposed. A proof‐of‐the‐concept TADF emitter with 3,6‐diphenyl‐9*H*‐carbazole (D) connected at the 3’3‐positions of 9*H*‐xanthen‐9‐one (A), the void carbon‐atom with no distribution of the highest occupied molecular orbital (HOMO) of A‐segment, realizes more efficient and blue‐shifted emission compared with the contrast D‐A isomers. The deeper HOMO‐2 of A is found to participate into conjugation rather than HOMO, providing a wider‐energy‐gap. The corresponding blue device exhibits a *y* color coordinate (CIE*_y_*) of 0.252 and a maximum external quantum efficiency of 27.5%. The stability of this compound is further evaluated as a sensitizer for a multiple resonance fluorophore, realizing a long lifetime of ≈650 h at an initial luminance of 100 cd m^−2^ with a CIE*_y_* of 0.195 and a narrowband emission with a full‐width‐at‐half‐maxima of 21 nm.

## Graphical abstract

An advanced donor‐void‐acceptor motif is proposed to construct blue thermally activated delayed fluorescent molecular, realizing a unprecedently high maximum external quantum efficiency of ≈27.5% as emitter and a long LT95 (time decays to 95% of the initial luminance of 100 cd m^−2^) of ≈650 h with a full‐width‐at‐half‐maxima of 21 nm (*y* color coordinate of 0.195) as sensitizer.

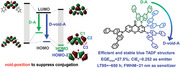

## 1 Introduction

Organic light‐emitting diodes (OLEDs) have made a successful leap from laboratory to large‐scale industrial production as displays for smart phones and televisions.^[^ ^1^ ^]^ This success essentially lies in the ability of emitters to harvest all excitons formed under electrical excitation with long operation stability. Nevertheless, highly efficient and stable blue devices remain a formidable challenge and the OLED industry still relays on inefficient conventional fluorescent emitters for blue emission.^[^ ^2^, ^3^ ^]^ Finding new emitting mechanisms thereof has been an exigent task for researchers in this fields.^[^ ^4^, ^5^, ^6^ ^]^ One of the most promising strategies is the utilization of materials with thermally activated delayed fluorescence (TADF), whereby otherwise lost dark triplet excitons can be up‐converted into the emissive singlets via efficient reverse intersystem crossing induced by small singlet‐triplet energy gap (Δ*E*_ST_).^[^ ^7^, ^8^, ^9^ ^]^ After the pioneering works of Adachi et al., TADF emitters have witnessed a rapid development.^[^ ^10^, ^11^, ^12^, ^13^ ^]^ Particularly, highly efficient blue OLEDs have preliminarily achieved utilizing blue TADF compounds as emitters or sensitizers, though their lifetimes are still far from the required lifetime of the industry,  realistically a T95 (time to 95% of the initial luminance) of 5000 h.^[^ ^14^ ^]^

**Scheme 1:**
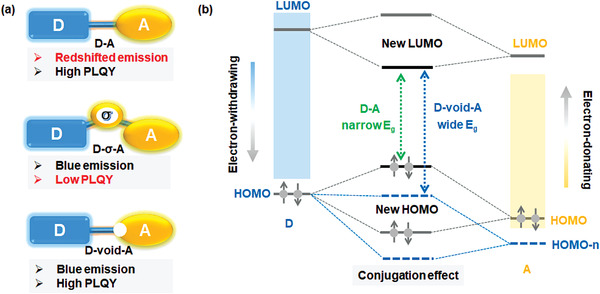
a) D‐A, D‐*σ*‐A, and D‐void‐A type TADF molecules. b) The conjugation effect between D‐A units that leads to redshifted emission.

A small Δ*E*_ST_ is essential for the TADF process, which usually took place from a charge transfer (CT) excited sates in molecules with a donor–acceptor (D‐A) structure. The highest occupied molecular orbital (HOMO) and the lowest unoccupied molecular orbital (LUMO) are spatially separated on the D and A groups, respectively.^[^ ^15^ ^]^ On the basis of this molecular design concept, a number of D‐A type TADF materials have been developed and exhibited remarkable performances.^[^ ^16^, ^17^ ^]^ Nevertheless, owing to the strong electronic interactions between the D and A moieties, these D‐A motifs always lead to clear redshifted emissions, making it difficult to achieve blue emission.^[^ ^14^ ^]^ Specifically, both inducive effect and conjugative effects exist between D and A groups, as illustrated in **Scheme** **1**. The inductive effect involves the electron‐withdrawing ability and electron‐donating property of A and D, which will deepen the HOMO level of donors or shallow the LUMO level of A, facilitating larger energy gaps. On the contrary, the conjugative effect, whereby the molecular orbitals of D and A parts are conjugated to new molecular orbitals that are more delocalized and therefore generally lower in energy, usually narrow the energy gap between HOMO of D and LUMO of A. Commonly, the conjugation effect is much stronger than the inductive one, thus always causing redshifted emission. An effective strategy to suppress the D and A interactions is the adoption of nonconjugated *σ*‐linker to construct molecules with D‐*σ*‐A motif. ^[^ ^18^, ^19^ ^]^ However, the *σ*‐conjunction conversely leads to the negligible HOMO–LUMO overlap for near‐zero oscillator strength (*f*) and also brings flexibility to the molecular backbone to induce significant nonradiative decay, consequently leading to unsatisfied both photoluminescent (PL) and electroluminescent (EL) performances. Therefore, the most common blue TADF emitters still relay on the utilization of donors with deep HOMO levels and/or acceptors with shallow LUMO levels to compensate the energy loss from conjugation effect,^[^ ^20^, ^21^ ^]^ making it hard to balance the emitting color and efficiency. What is more, the situation is even tough when take long‐term operation stability under EL into consideration, given that only quite few blue TADF emitters have exhibited decent stability albeit substantial efforts.^[^ ^14^ ^]^ In this regard, more advanced molecular design strategies that give consideration to both color purity and stability are ongoing pursuit.

Herein, we proposed an advanced D‐void‐A motif for the goal of highly efficient and stable blue TADF emitters. By modulating the linkage topology of isomers with 9*H*‐xanthen‐9‐one (Xo) as A while 3,6‐diphenyl‐9*H*‐carbazole (PhCz) as D, it is unveiled that when D and A groups being connected by the void‐position in the *π*‐backbone of A, that is the carbon (C)‐atom with nondistribution of A‐segment HOMO, the conjugation between HOMO levels of donor and acceptor can be suppressed. Instead, a much deeper HOMO‐2 level of A, in this case, will participate into forming the new HOMO level of the whole molecular, giving birth to a wider energy gap for blue emission as illustrated in Scheme 1 and a large *f* value meanwhile. The corresponding emitter with PhCz connected on the 3,3'‐positions (the void carbon atom) of Xo realizes a photoluminescence quantum yield (PLQY) of 92% with an emission peak at ≈440 nm in toluene, which is significantly blue‐shifted compared with the reference donor–acceptor type isomers. The corresponding device exhibits a CIE*_y_* of 0.252 and a maximum external quantum efficiency (EQE_max_) of 27.5%, which was a record‐high value for previously reported TADF emitters based on Xo groups. We further evaluated the EL stability of this D‐void‐A compound by using it as a TADF sensitizer for a multiple resonance fluorophor, realizing a long device LT95 (time required to decay to 95% of the initial luminance) of ≈650 h at an initial luminance of 100 cd m^−2^ with a CIE*_y_* of 0.195 and a narrowband blue emission with a full‐width‐at‐half‐maxima (FWHM) of 21 nm simultaneously. The lifetime is among the highest at this specific color, validating the good stability of Xo unit as donor in TADF emitters for the first time. Aside from offering an advanced D‐void‐A structure for highly efficient and stable blue TADF emitters, this work here also sheds new light on manipulating linkage topology of isomers based on Xo groups, which will bring large molecular design flexibility for Xo‐based TADF emitters.

## 2 Results and Discussions

**Figure 1:**
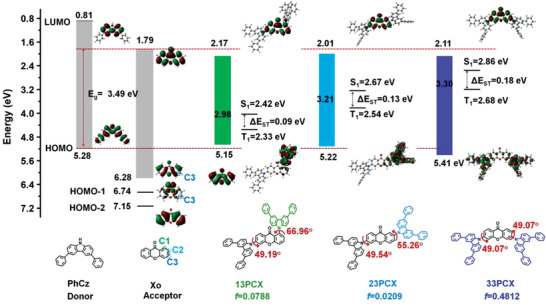
The calculated geometry and electronic properties of 13PCX, 23PCX, and 33PCX, respectively.

Featuring large plane structure, the electron‐deficit Xo group should naturally facilitate high radiative decay, which has been adopted in our previous works and others’, exhibiting excellent device performances.^[^ ^22^, ^23^, ^24^ ^]^ Additionally, the C1, C2, and C3 positions of Xo are feasible for substitution, rendering large molecular design flexibility. The electronic properties of Xo group were calculated based on density functional theory (DFT) with B3LYP/ 6–31G(d,p) basis set with its optimized conformation at the ground state. It is worth noting that there is limited HOMO distribution on the C3 atom of Xo plane as illustrated in **Figure** **1**. Interestingly, HOMO‐1 of Xo also showed a void C3 atom while HOMO‐2 exhibited clear distribution on C3. Considering the fact that the conjugation interaction requires orbital overlap, C3 atom thereof can be taken as one void position for the conjugation between HOMOs of D and A groups. To further clarify the void nature, we used Mulliken population method to describe the distribution of C1, C2, and C3 in HOMO of Xo.^[^ ^25^ ^]^ In this method, cross terms are divided equally by basis functions in 6–31G(d,p). We found the distribution of C1 and C2 is 4.65% and 12.77%, respectively, as shown in Figure S1 in the Supporting Information. As comparison, the distribution of C3 is only 0.40%, which means C3 has very little distribution of HOMO of Xo. In terms of the D group, PhCz was chosen. Previous reports usually adopted donors with high steric effect for Xo to obtain small Δ*E*_ST_, including 1,3,6,8‐tetramethyl‐9*H*‐carbazole, 9,9‐dimethyl‐9,10‐dihydroacridine, etc., which, however, induce large dihedral angles for a small *f*.^[^ ^26^, ^27^, ^28^ ^]^ Here, the less steric effect between PhCz and Xo groups favors moderate dihedral angles to keep a large *f* while the delocalized HOMO into the two substituted phenyl rings facilitates a small Δ*E*_ST_. The frontier energy levels of both groups were calculated to be 1.789 and 5.238 eV for Xo LUMO and PhCz HOMO, respectively. A large energy gap (*E*_g_) of 3.449 eV thereafter can be calculated, facilitating blue emission. To avoid the influence of different HOMO distribution, we further stimulated the molecules with only one donor as illustrated in Figure S2 in the Supporting Information. Clearly, a similar phenomenon was observed that 3PCX showed deeper HOMO energy level than PhCz while 1PCX and 2PCX showed shallower ones. Those results should further validate our inspiration about D‐void‐A structure.

To validate the role of the void C3 atom, the optimal geometry conformation and electronic properties of three isomers, 1,6‐bis(3,6‐diphenyl‐9*H*‐carbazol‐9‐yl)‐9*H*‐xanthen‐9‐one (13PCX), 2,6‐bis(3,6‐diphenyl‐9*H*‐carbazol‐9‐yl)‐9*H*‐xanthen‐9‐one (23PCX), and 3,6‐bis(3,6‐diphenyl‐9*H*‐carbazol‐9‐yl)‐9*H*‐xanthen‐9‐one (33PCX), were calculated for comparison, as illustrated in Figure 1. Here, this two‐donor motif was adopted for Xo to further delocalize HOMO distributions. Owing to the different steric effect, different dihedral angels between Xo and PhCz attached on different carbon positions were recorded, being 66.96° for C1 in 13PCX, 55.26° for C2 in 23PCX while 49.07° for C3 in 33PCX, respectively. Owing to those twisted structure, for all three compounds, the LUMOs are mainly resided on the Xo groups while the HOMOs mainly on PhCz units. Meanwhile, the former is slightly extended to PhCz groups while the later to the Xo moieties, suggesting that both Xo and PhCz groups participate into the formation of molecular orbitals. The calculated energy levels of those compounds were also provided, all exhibiting deeper LUMO energy levels than those of Xo and PhCz groups. Intriguingly, a different situation was observed for the HOMO parts. For 13PCX and 23PCX, their HOMO energy levels are shallower than those of PhCz and Xo groups while 33PCX contrarily shows a deeper HOMO level compared with that of PhCz. As aforementioned, both conjugation and inductive effects exist between D and A groups; the former will shallower HOMO or deeper LUMO levels by forming the new orbitals while the latter being just the reverse. Therefore, it can be explained by the stronger conjugation effect than the inductive effect that the deeper LUMO levels of all three compounds and the shallower HOMOs of 12PCX and 23PCX compared with the respective ones of D and A groups.

Nevertheless, a different situation was observed for the HOMO level of 33PCX. Simplified, the conjugation effect requires the interactions between HOMOs of D and A segments and thus overlap between them should be satisfied. Since the HOMO of A distributes on its C1 and C2 positions, the interactions between HOMOs of D and A groups in 13PCX and 23PCX can be expected. On the contrary, no HOMO of A distributes on its C3 position and the same situation was observed for the HOMO‐1. Therefore, both HOMO and HOMO‐1 cannot participate in the D‐A conjugation. In sharp contrast, HOMO‐2 of A will interact with HOMO of D to form the new molecular orbital. Since HOMO‐2 is much deeper than the HOMO level, the new molecular orbital should also be deep as illustrated in Scheme 1. Thus, 33PCX exhibits a deeper HOMO level than the other two compounds. Consequently, a wide energy gap of 3.30 eV for 33PCX was obtained, larger than those of 13PCX (2.98 eV) and 23PCX (3.21 eV). We have to point out that besides the conjugation effect, inducive effect may also lower the HOMO energy levels. However, it is difficult to distinguish those two interactions at this moment. For 13PCX and 33PCX, the inducive effect should be similar but rather different energy levels, which may reflect the great influence of conjugation effect and validate our inspirations that it is the HOMO‐2 of acceptor participates in the conjugation. Yet, more efforts are still required to clarify this debate.

The state energy was also predicted using time‐dependent (TD)‐DFT with the same basis, revealing a large singlet energy of 2.86 eV for 33PCX while only 2.42 and 2.67 eV for 13PCX and 23PCX, respectively. Besides, small Δ*E*_ST_s of 0.090, 0.128, and 0.181 eV for 13PCX, 23PCX, and 33PCX were also obtained owing to the spatially separated distributions of frontier molecular orbitals, which favor efficient TADF properties. What is also worth noting is that a large *f* = 0.4812 was obtained for 33PCX, significantly higher than those of 13PCX and 23PCX, which were only 0.0788 and 0.0209, respectively. The reason should arise from, on the one hand, the more delocalized HOMO distribution on both PhCz units of 33PCX, on the other hand, the relatively larger HOMO–LUMO overlap due to the symmetrical molecular structure. The high *f* value should facilitate efficient radiative decay process of 33PCX. To the best of our knowledge, the D‐*σ*‐A molecules always feature near‐zero *f* values owing to the negligible HOMO–LUMO overlap.^[^ ^29^ ^]^ The D‐void‐A motif of 33PCX here unveiled that by involving low energy HOMO‐2 into conjugation rather than the HOMO, not only a large energy gap for blue emission but also decent *f* for efficient radiative decay can be obtained to maximize the emitter performances. Such D‐void‐A motif thereof possesses the viability to outperform D‐A and D‐*σ*‐A structure to construct blue TADF emitters. Interestingly, with the same donor and acceptor group, both D‐A and D‐void‐A structure can be obtained, pointing out the crucial importance in manipulating the linkage topology of D and A units to modulating compound properties.

**Figure 2:**
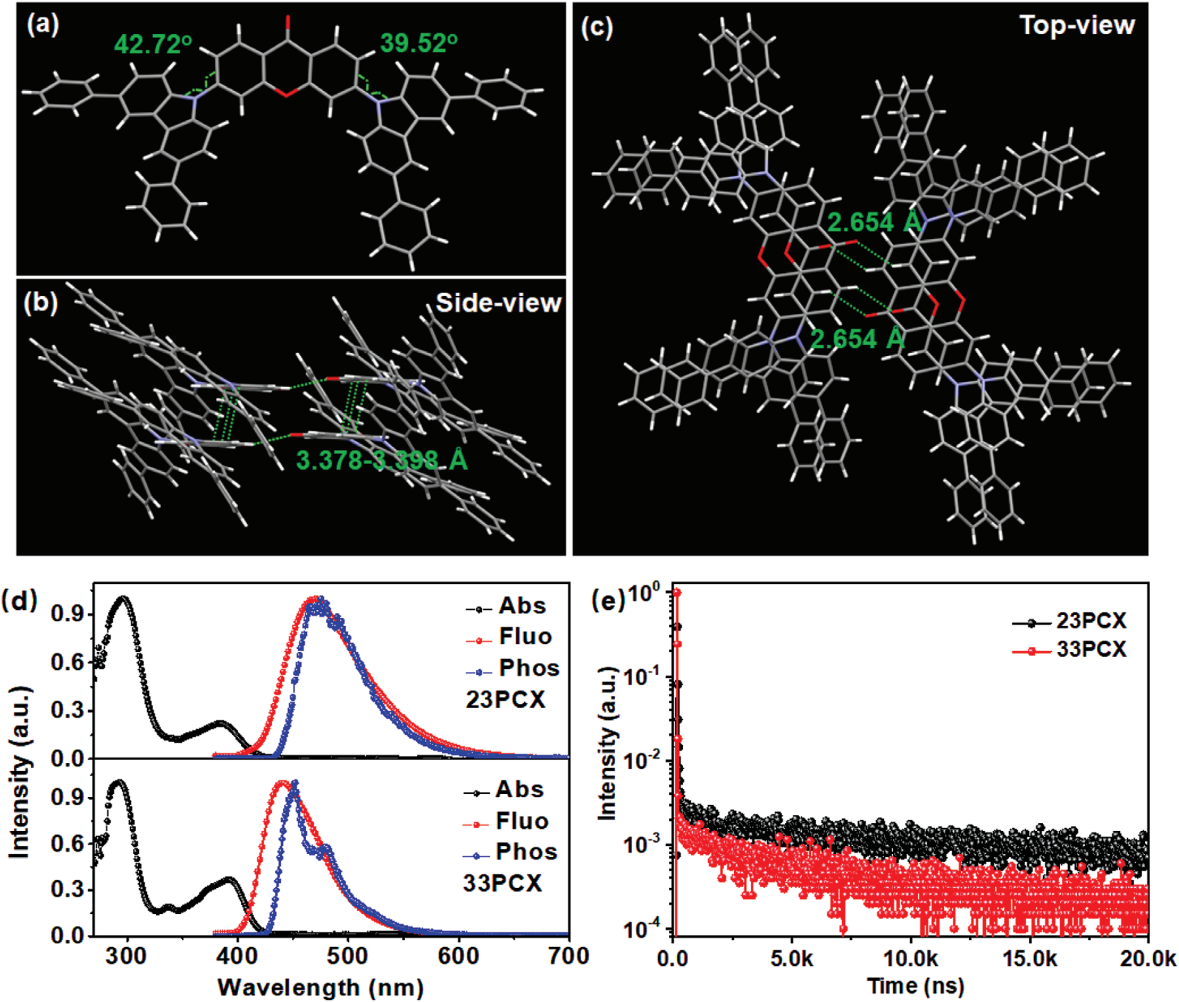
a) The single‐crystal structure of 33PCX. The single‐crystal packing mode of 33PCX observed from b) side‐view and c) top‐view. d) The absorption, fluorescence, and phosphorescence emission spectra of 23PCX and 33PCX in toluene with a concentration of 10^–5^ m. e) The PL decay curves of PPF‐doped films recorded at the emission peaks of the emitters.

On the basis of the calculation results above, 23PCX and 33PCX possess the potential for blue emission and thus those two emitters were synthesized as shown in Schemes S1 and S2 in the Supporting Information. The two compounds were facilely synthesized by Buchwald–Hartwig coupling reaction with high yields, which were then carefully characterized by NMR, mass spectra, and elemental analysis, respectively. We obtained the single crystal of 33PCX during the sublimation process and the crystal structure was thereof analyzed. A twisted structure with dihedral angels of 42.72° and 39.52° between D and A groups was recorded as illustrated in **Figure** **2**a. The molecular packing structures were also provided from the side view (Figure 2b) and top view (Figure 2c). Strong hydrogen bonds with identical instance of 2.654 Å were observed between the oxygen atom of carbonyl group in one molecule and the hydrogen atom next to the carbonyl group of another molecule. Besides, *π*–*π* interaction stacking between Xo groups was observed with instances in the range of 3.378–3.398 Å. Those multiple molecular interactions render a packing mode with Xo groups packing inside while PhCz moieties outside, which should facilitate both electron and hole transporting channels.

Figure 2d depicts the absorption (Abs) and fluorescence (Fluo) spectra of 23PCX and 33PCX in toluene solution with concentration of 10^–5^ m. Both compounds showed strong absorption peaks about 400 nm, assigned to the intramolecular charge transfer (CT) transition from PhCz to Xo. Interestingly, it was noticed that 23PCX showed a CT absorption peak at 388 nm, being blue‐shifted compared with that of 33PCX (393 nm). On the contrary, in terms of the optical band gap (*E*_g_), 23PCX showed a relatively narrower *E*_g_ of 2.88 eV than 33PCX (2.97 eV), obtained from the onset of CT absorption edge. We also calculated the theoretical emission energy of 23PCX and 33PCX, being 2.36 and 2.62 eV, respectively, which is in agreement with the order of *E*_g_.^[^ ^30^ ^]^ Such phenomenon has also been observed in previous works.^[^ ^14c^ ^]^ Those CT transitions consequently lead to featureless wide emission spectra peaking at 470 nm for 23PCX and 440 nm for 33PCX. The blue‐shifted emission of 33PCX is in corresponding with the theoretical results owing to the large energy gap from the D‐void‐A structure. The phosphorescence (Phos) emissions of 23PCX and 33PCX were also recorded under 77 K. To reveal the excited nature of triplet states, the natural transition orbitals analysis was conducted and provided in Figure S3 in the Supporting Information. Clearly, the hole and particle distributions are spatially separated, indicating the main CT characters of the T_1_ states of both compounds. The broad featureless Phos‐spectrum of 23PCX evidences the CT property of its T_1_. In the case of 33PCX, a shoulder peak was observed for its Phos‐spectrum, suggesting this emission may combine both CT and localized (LE) characters. We thereafter obtain the energies of both T_1_ states of 23PCX and 33PCX from the onsets of their phosphorescent spectra, being 2.83 and 2.88 eV, respectively, as illustrated in **Table** **1**. Their Δ*E*_ST_s can be thereafter obtained, being 0.12 eV for 23PCX and 0.16 eV for 33PCX. The relatively large Δ*E*_ST_ of 33PCX should arise from its more significant HOMO–LUMO overlap as aforementioned.

**Table 1:** The photophysical and electrochemical properties of emitters

Emitters	S_1_ ^a)^ [eV]	T_1_ ^a)^ [eV]	Δ*E*_ST_ ^a)^ [eV]	HOMO [eV]	LUMO [eV]	*Φ*_P_/*Φ*_D_^b)^	*τ*_P_ ^b)^ [ns]	*τ*_D_ ^b)^ [µs]	*k*_r_ ^b)^ [10^7^ s^−1^]	*k*_RISC_ ^b)^ [10^5^ s^−1^]
23PCX	2.95	2.83	0.12	5.5	3.0	0.19/0.69	17.9	5.74	1.1	8.0
33PCX	3.04	2.88	0.16	5.7	2.9	0.41/0.51	14.7	5.24	2.8	4.3

^a)^ Measured in toluene (10^–5^ m);

^b)^ Values obtained from the PPF: 20 wt% dopant films.

We further measured that TADF characters of both emitters by dispersing them into a wide energy gap host, 2,8‐bis(diphenylphosphoryl)dibenzo[*b,d*]furan (PPF) with a concentration of 20 wt%. As illustrated in Figure S4 in the Supporting Information, both films showed wide structureless emission from dopants with peaks of 472 and 485 nm for 23PCX and 33PCX, respectively. The PL transient decay curves of the doped films are provided in Figure 2e. Clear prompt parts with lifetimes of 17.9 and 14.7 ns as well as delayed components with lifetimes of 5.74 and 5.24 µs were recorded for 23PCX and 33PCX, respectively, evidencing the existence of TADF emission. The PLQYs of the two films were also measured to be 0.88 for 23PCX and 0.92 for 33PCX with the prompt parts of 0.19 and 0.41, respectively. The radiative decay rate (*k*_r_) thereof can be calculated to be 1.1 × 10^7^ s^−1^ for 23PCX and 2.8 × 10^7^ s^−1^ for 33PCX. The much higher *k*_r_ of 33PCX should arise from its large *f* value as aforementioned. The *k*_RISC_ of both compounds were also obtained and 33PCX exhibited a moderate *k*_RISC_ of 4.3 × 10^5^ s^−1^, which is relatively lower than that of 22PCX (8.0 × 10^5^ s^−1^). The reason should be attributed to the relatively larger Δ*E*_ST_ of 33PCX. Those results here testify that different to previously reported D‐*σ*‐A structure, D‐void‐A motif favors not only blue emission but also high PL performances. The concentration‐dependent TADF characteristics of 23PCX and 33PCX are also provided in Figure S4 and Table S1 in the Supporting Information.

The oxidation and reduction potentials of the two compounds in the dichloromethane and *N*,*N*‐dimethylformamide (DMF) solution using cyclic voltammetry (CV) were also measured, respectively. Both compounds showed quasi‐reversible/ reversible oxidation/ reduction potentials as provided in Figure S5 in the Supporting Information, suggesting the good electrochemical stability of both donor and acceptor moieties. The frontier energy levels can be thereof obtained, with LUMO levels being 3.0 and 2.9 eV while HOMO levels being 5.5 and 5.7 eV for 23PCX and 33PCX, respectively. Bearing the same D and A units, the difference in energy levels should arise from the different D/A interactions owing to the varied liking topology. The evidently deeper HOMO energy of 33PCX than that of 23PCX agrees well with the calculated results above, which should arise from not only the suppressed conjugation effect by the D‐void‐A motif.

**Figure 3:**
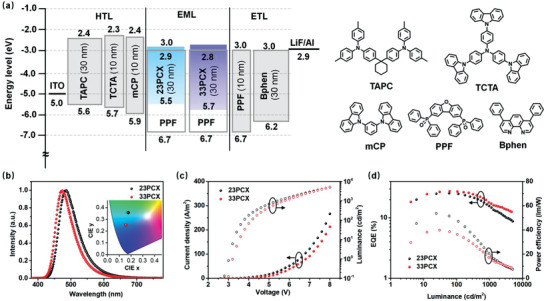
a) The energy levels of devices and the molecular structures. b) The EL spectra of devices recorded at 1000 cd m^−2^. c) The current–density–voltage–luminance curves of devices. d) The EQE–luminance and power efficiency–luminance curves of devices.

To evaluate the EL performances of the two emitters, devices with structures of indium tin oxide (ITO)/TAPC (30 nm)/TCTA (10 nm)/mCP (10 nm)/PPF: 20 wt% emitters (30 nm)/PPF (10 nm)/Bphen (30 nm)/LiF (0.5 nm)/Al (150 nm) were fabricated. Here, TAPC, TCTA, mCP, and Bphen stand for 1,1‐bis[4‐[*N,N′*‐di(*p*‐tolyl)amino]phenyl]cyclohexane, 4,4′,4′‐*tris*(carbazol‐9‐yl)‐triphenylamine, 1,3‐di‐9‐carbazolylbenzene, and 4,7‐diphenyl‐1,10‐phenanthroline, respectively. The energy levels of devices and the molecular structures are provided in **Figure** **3**a. Owing to the deep HOMO energy level of PPF, it is reasonable to anticipate that charge recombination should directly happens on the dopants. The pristine layers of high‐triplet‐energy mCP and PPF were adopted to confine excitons on the dopants. Figure 3b exhibits the EL spectra of 23PCX and 33PCX‐based devices recorded at 6 V with emission peaks of 484 and 469 nm, corresponding to CIE coordinates of (0.176, 0.357) and (0.156, 0.252), respectively, as illustrated in **Table** **2**. Their EL spectra agreed well with the PL ones, validating that D‐void‐A structure favors blue emission. The current density–voltage–luminance characteristics are given in Figure 3c, revealing the operation voltages of 3.02, 3.79, and 5.48 V as well as 3.19, 4.17, and 5.73 V for 23PCX and 33PCX at luminance of 10, 100, and 1000 cd m^−2^, respectively. The relatively larger operation voltage for device based on 33PCX than the one with 23PCX should be assigned to the relatively large energy gap of 33PCX, which hinders charge injection from the adjacent layers compared with 23PCX. The EQE–luminance–power efficiency curves are depicted in Figure 3d. Benefiting the higher PLQY of 33PCX than 23PCX, the device using it as emitter showed a relatively higher EQE_max_ of 27.5%, which remained 27.3% at 100 cd m^−2^ and 18.3% at 1000 cd m^−2^. The device with 23PCX showed an EQE_max_ of 25.5%, remaining at 25.0% and 15.7% under luminance of 100 and 1000 cd m^−2^, respectively. The results are rather interesting that 33PCX realized higher EQEs with even blue‐shifted emission. To the best of knowledge, the EQE_max_ of 33PCX here represents a record‐high one among the previously reported TADF emitters based on Xo as acceptor. In addition, high maximum power efficiencies (PE_max_s) of 53.5 and 39.7 lm W^−1^ were observed for 23PCX and 33PCX‐based devices. The relatively higher PE_max_ of 23PCX should arise from its lower operation voltages due to its narrower energy gap. Interestingly, previously reported TADF molecules with Xo segment as donor were exclusively connected by its 3,3'‐positions,^[^ ^22^, ^23^, ^24^ ^]^ lacking comprehensive understanding on isomer structures. Our work here validates the excellent performances of 23PCX, suggesting that different molecular topologies also deserve to be considered for Xo‐based TADF emitters. The thereof increased molecular design flexibility may shed new light on more efficient TADF molecules based on Xo group, regardless of the emitting colors.

**Table 2:** Summary of device performances

Device	Voltage [V]			EQE [%]			PE [lm W^−1^]			CIE (*x*, *y*)^c)^
	10 cd m^−2^	100 cd m^−2^	1000 cd m^−2^	Max	100 cd m^−2^	1000 cd m^−2^	Max	100 cd m^−2^	1000 cd m^−2^	
23PCX^a)^	3.02	3.79	5.48	25.5	25.0	15.7	53.5	48.1	20.5	(0.176,0.357)
33PCX^a)^	3.19	4.17	5.73	27.5	27.3	18.3	39.7	36.2	17.2	(0.156, 0.252)
23PCX^b)^	2.92	3.45	4.54	25.1	21.6	23.4	40.4	38.9	29.9	(0.149, 0.241)
33PCX^b)^	2.94	3.49	4.67	20.6	13.1	20.2	24.1	19.3	21.7	(0.140, 0.195)

^a)^ TADF OLEDs;

^b)^ TSF OLEDs;

^c)^ Recorded at 1000 cd m^−2^.

**Figure 4:**
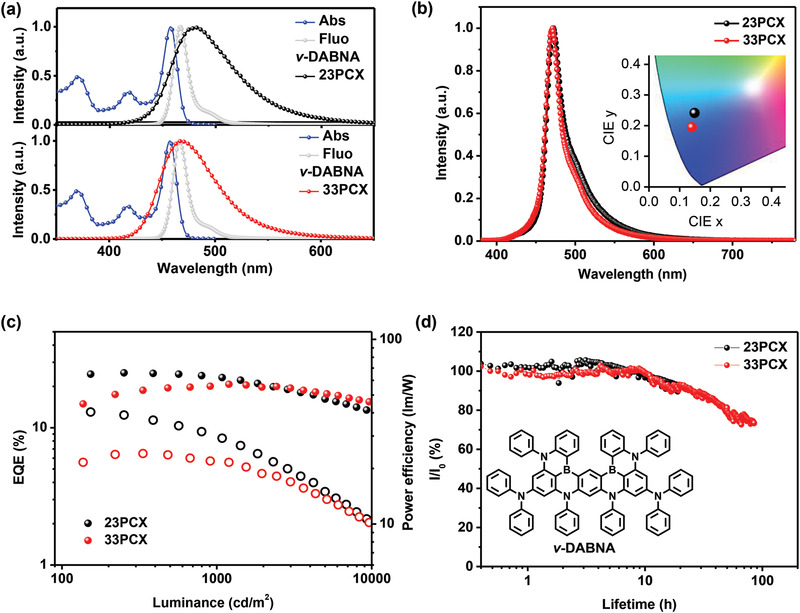
a) The absorption and emission spectra of v‐DABNA in toluene as well as the emission spectra of 23PCX and 33PCX. b) The EL spectra of v‐DABNA based on devices with mCPBC as the host. c) The EQE–brightness (closed circle) and power efficiency–brightness (open circle) characters of *ν*‐DABNA based on devices sensitized by 23PCX and 33PCX. d) The luminance decay curves of the same devices used in (c), which are recorded at an initial luminance of 1000 cd m^−2^. Inset shows the chemical structure of *ν*‐DABNA.

Aside from high efficiency, a good operation stability is equally important for the real applications of blue TADF molecules. Rather than being utilized as emitters, TADF molecules as sensitizers for fluorescent dopants, known as TADF‐sensitized fluorescence (TSF) or hyperfluorescence,^[^ ^31^, ^32^ ^]^ have been proved more chance to break the mutual exclusion of high efficiency and stability of blue OLEDs. Particularly, when multiple resonance (MR) fluorophors were adopted as the final emitters, not only narrowed emission spectra width for high color purity but also extended device operation lifetimes have been observed.^[^ ^33^, ^34^, ^35^, ^36^ ^]^ Inspired by previous works, we further evaluated the EL stabilities of those two emitters as sensitizers for a reported blue MR emitter, *N^7^*,*N^7^*,*N^13^*,*N^13^*,5,9,11,15‐octaphenyl‐5,9,11,15‐tetrahydro‐5,9,11,15‐tetraaza‐19b,20b‐diboradinaphtho[3,2,1‐*de*:1’,2’,3’‐jk]pent‐acene‐7,13‐diamine (*v*‐DABNA),^[^ ^37^ ^]^ with a proved stable device structure of ITO/HATCN (5 nm)/NPB (30 nm)/TCTA (10 nm)/mCPBC: 30 wt% sensitizers: 1 wt% *v*‐DABNA (30 nm)/CzPhPy (10 nm)/DPyPA:Liq (1:1, 30 nm)/LiF (0.5 nm)/Al (150 nm).^[^ ^38^ ^]^ Here, HATCN, NPB, mCPBC, CzPhPy, DPyPA, and Liq represent dipyrazino[2,3‐f:2’,3’‐h]quinoxaline‐2,3,6,7,10,11‐hexacarbonitrile, *N*,*N*′‐bis(1‐naphthalenyl)‐*N*,*N*′‐diphenyl‐[1,1’‐biphenyl]‐4,4’‐diamine, 9‐(3‐(9*H*‐carbazol‐9‐yl)phenyl)‐9*H*‐3,9′‐bicarbazole, 4,6‐bis(3‐(9*H*‐carbazol‐9‐yl)phenyl) pyrimidine, 9,10‐bis(6‐phenylpyridin‐3‐yl)anthracene, and (8‐quinolinolato)lithium, respectively. *v*‐DABNA has been demonstrated to be an efficient terminal emitter with high PLQY of ≈100% and narrowband emission with an FWHM of 14 nm in toluene, as illustrated in **Figure** **4**a. Owing to the extremely small stokes shift (9 nm) of dopant and wide emission band of sensitizers, clear dopant absorption‐sensitizer emission spectra overlap was observed even though dopant possesses a relatively blue‐shifted emission peak. Compared with 23PCX, the blue‐shifted emission of 33PCX should favor more efficient energy transfer as clearly evidenced by the absorption–emission overlap in Figure 4a. The ability of Förster energy transfer (FRET) from sensitizer to dopant can be quantified using the FRET radius (*R*_0_), defined as the intermolecular distance at which the FRET rate constant is equal to the total decay rate constant of the donor in absence of the acceptor. The *R*_0_ can be expressed as

(1) $$R_{0}^{6}=\phi_{D}\kappa^{2}\left(\frac{9000(\ln10)}{128\pi^{5}N_{A}n^{4}}\right)\int_{0}^{\infty }F_{D}\left(\lambda \right)\varepsilon_{A}\left(\lambda \right)\lambda^{4}d\lambda$$

In this equation, *κ* ^2^ is usually assumed to be 2/3 For a random distribution of donor–acceptor pairs, while *n* is about 1.7 for most of the organic materials. A larger *R*_0_ of 3.8 nm for 33PCX was obtained than that of 3.3 nm for 23PCX, evidencing the more efficient energy transfer from 33PCX to *v*‐DABNA.

Figure 4b provides the EL spectra of those two devices, providing FWHMs of 24 and 21 nm for 23PCX and 33PCX‐based devices, respectively, with identical emission peaks of 471 nm. The relatively wider emission band of 23PCX device than that of 33PCX device should arise from the residual emission from 23PCX. CIE coordinates of (0.149, 0.241) for 23PCX device and (0.140, 0.195) for 33PCX device were recorded. Figure 4c illustrates the EQE–luminance characters of those devices, revealing a maximum EQE of 25.1% and 20.6% for 23PCX and 33PCX, respectively. It is interesting to note that, as illustrated in Figure S6 in the Supporting Information, with this device structure, both 23PCX and 33PCX showed relatively higher EQEs and narrower FWHMs as sensitizer than as TADF emitters. Besides, TSF device based on 23PCX showed relatively higher efficiency than the one based on 33PCX. One plausible reason should be attributed to the more efficient reverse intersystem crossing (RISC) process of 23PCX in mCPBC as illustrated in Table S1 in the Supporting Information. Previous works have demonstrated rather than a high FRET radius, the *k*_RISC_ of the sensitizer matters more in determining the performances of the sensitized devices.^[^ ^35^ ^]^ Also, we noticed that for devices with mCPBC as host, 33PCX as emitter showed relatively lower EQE than 23PCX, which is contrary to the situation in PPF. One plausible reason should be the relatively low polarity of mCPBC, which is not beneficial to reduce the Δ*E*_ST_ of 33PCX and promote RISC process. Such host–guest interactions have been thoroughly studied and adopted to optimize the efficiency of TADF devices.^[^ ^39^ ^]^ Other parameters may also account for this phenomenon including the relatively lower triplet state energy of mCPBC and the different carrier transport ability as mCPBC is a hole transport dominating host while PPF is an electron one.

In addition, Figure 4d displays the luminance of those OLEDs as a function of operational time under constant current density at an initial luminance (*L*_0_) of 1000 cd m^−2^. LT95s of ≈13 and ≈15 h were recorded for devices with 33PCX and 23PCX, respectively. The LT95 of those devices at an initial luminance of 100 cd m^−2^ can be extrapolated using the equation for $\mathrm{LT}95\left(100\;\;\mathrm{cd}\;m^{-2}\right)=\mathrm{LT}95\left(1000\;\;\mathrm{cd}\;m^{-2}\right)\times {\left(\frac{1000\;\mathrm{cd}m^{-2}}{100\;\mathrm{cd}m^{-2}}\right)}^{n}$ with a degradation acceleration factor (*n*) of 1.7, resulting in ≈650 h for 33PCX and ≈750 h for 23PCX‐based devices. Interestingly, the device based on 33PCX bearing even bluer emission exhibited similar LT95 compared with the one with 23PCX. Recently, substantial efforts have been devoted on improving the operation lifetimes of devices based on *v*‐DABNA as emitter by developing stable TADF sensitizers. And the LT95s of ≈18 h and <10 h at initial luminance of 1000 cd m^−2^ have been obtained by Adachi's group and Lee's group, respectively, for bottom‐emissive single‐unit devices,^[^ ^33^, ^34^ ^]^ corresponding to LT95s of ≈902 and ≈500 h assuming the same degradation acceleration factor. The LT95s of our OLEDs are comparable or even better than those champion results, validating the excellent stability of our blue emitters, which may be even promoted by further optimizing device structure. Moreover, to the best of our knowledge, it is the first time that the EL operation stability of Xo‐based TADF compounds were evaluated. Given the fact that phenyl ring‐substituted carbazole groups have demonstrated good stability,^[^ ^40^, ^41^ ^]^ the long operation lifetimes here testify that Xo group is also stable as donor in TADF compounds. Different to the substantial stable donors, the acceptors that exhibited good long‐term stability are still rare. To the best of our knowledge, till now only some cyano‐ and triazine‐derivatives have exhibited decent operation stability,^[^ ^9^, ^20^, ^33^, ^34^ ^]^ greatly limiting the design flexibility of TADF molecules. Our work here proves the good stability of Xo group as acceptor, which will motivate further researches on more stable full color TADF emitters‐based this unit. What should be further pointed out is that though the red‐shifted emission, 23PCX also exhibited good performances as sensitizer with even higher efficiency, further validating the great potential of manipulating the molecular topology for Xo‐based compounds, which has been neglected previously.

## 3 Conclusion

In conclusion, by analyzing three isomers based on PhCz donor and Xo acceptor, we found that a D‐void‐A structure can be formed when PhCz was connected to the Xo group through C3‐position where negligible HOMO distribution of Xo located. In this scenario, the conjugation between HOMOs of donor and acceptor can be suppressed and instead a much deeper HOMO‐2 of acceptor will participate into forming the new HOMO level of whole molecule, resulting in a wider energy gap for blue emission and a large *f* value at the same time. The proof‐of‐the‐concept molecule 33PCX realized a high PLQY of 92% with an emission peak at ≈440 nm in toluene, which showed clear blue‐shifted emission compared with the reference D‐A type isomers. The corresponding device exhibited an EQE_max_ of 27.5% with an CIE*_y_* of 0.252, which remained 27.3% at 100 cd m^−2^ and 18.3% at 1000 cd m^−2^. Furthermore, by adopting this D‐void‐A compound as sensitizer for a multiple resonance fluorophore, a long LT95 of >650 h at an initial luminance of 100 cd m^−2^ together with CIE*_y_* of only 0.195 and a small FWHM of 21 nm were recorded, first proving the good stability of Xo acceptor‐based TADF compounds. Our work here not only provides an advanced D‐void‐A structure for highly efficient and stable blue TADF emitters, but also validates the excellent performances of other isomers regardless of their red‐shifted emitting colors, suggesting that different molecular topologies should also be taken into consideration, which will bring large molecular design flexibility for full‐color TADF emitters based on Xo group.

## 4 Experimental Section

### General Information

All reagents were commercially available and being used without further purification. The experimental details on the synthesis and characterization of 23PCX and 33PCX are presented in the Supporting Information. A Bruker 600 MHz spectrometer was adopted for the ^1^H NMR spectra of the targeted compounds with the internal standard of tetramethylsilane (TMS). A Bruker Esquire ion‐trap mass spectrometer was used for the measurement of the compound mass spectra.

### Electrochemical Measurement

A Potentiostat/Galvanostat Model 283 (Princeton Applied Research) electrochemical workstation was used for the electrochemical measurements with Pt as the working electrode, platinum wire as the auxiliary electrode, and a Ag/AgCl system as the reference electrode standardized against ferrocene/ferrocenium. The reduction and oxidation potentials were measured in anhydrous DMF and dichloromethane solution, respectively, containing 0.1 m n‐Bu4NPF6 as supporting electrolyte with a scan rate of 100 mV s^–1^. And the HOMO and LUMO energies were calculated from the onset potential of oxidation and reduction curves, respectively.

### Single‐Crystals Characterization

The single crystal of 33PCX was obtained during the vacuum sublimation process and analyzed by a RIGAKU RAXIS‐RAPID diffractometer equipped with a graphite‐monochromator Mo‐K*α* radiation (*λ* = 1.54184 Å) at 173 K. The crystal structure was solved by direct methods and then refined with a full‐matrix least‐squares technique based on F2 with the SHELXL‐97 crystallographic software package.

[CCDC 2110406 contains the supplementary crystallographic data for this paper. These data can be obtained free of charge from The Cambridge Crystallographic Data Centre via www.ccdc.cam.ac.uk/data_request/cif.]

### Theoretical Calculations

Gaussian 09 program package was used to perform the geometrical and electronic properties of those emitters based on DFT using B3LYP with the 6–31G(d,p) atomic basis set in gas‐phase. TD‐DFT calculations were also conducted using B3LYP with the same basis set to evaluate the singlet and triplet state energies. The molecular orbitals were then visualized using Gaussview program.

### Optical Characterization of Organic Thin Films

An Agilent 8453 spectrophotometer was used to record the ultraviolet‐visible (UV‐vis) absorption spectra while an absolute PLQY measurement system (C9920‐02, Hamamatsu Photonics) was adopted to measure PLQYs in air atmosphere with an excitation wavelength of 360 nm. The samples were degassed prior to measurement. A transient spectrometer (Edinburg FL1000) was utilized for the measurements of PL spectra and PL transient decay curves of the doped films. A laser was used as the light source with a frequency of 50 Hz and the excitation wavelength was 360 nm. The solution concentration of molecules in toluene was 10^−5^ m for UV‐vis absorption and PL spectra measurements. All organic films were prepared on clean quartz substrates through spin‐coating by dichloromethane solution at room temperature.

The equations used to calculate the rate constant of TADF processes are as follows

(2) $$k_{\mathrm{PF}}=\frac{1}{\tau_{P}};\;k_{\mathrm{DF}}=\frac{1}{\tau_{D}};\;k_{\mathrm{ISC}}=\frac{k_{\mathrm{PF}}\phi_{\mathrm{DF}}}{\phi_{\mathrm{DF}}+\phi_{\mathrm{PF}}};\;k_{\mathrm{RISC}}=\frac{k_{\mathrm{DF}}k_{\mathrm{PF}}\phi_{\mathrm{DF}}}{k_{\mathrm{ISC}}\phi_{\mathrm{PF}}}$$

here, *k*_ISC_ is the intersystem crossing rate, *k*_RISC_ is the reverse intersystem crossing rate, *k*_PF_ is the prompt decay rate, and *k*_DF_ stands for the delayed decay rate, respectively. *ϕ*_P_ stands for the PLQY of the prompt part while *ϕ*_D_ for the delayed part.

### Device Fabrication and Measurement

All compounds used in this manuscript were twice purified by a vacuum sublimation approach. The ITO glass substrates were carefully precleaned before fabricating the corresponding devices. All devices were constructed under high vacuum with a pressure of 1 × 10^−5^ Pa. The thermally evaporated rates for all organic materials were in the range of 1.0–1.5 Å s^−1^. An absolute EQE measurement system (C9920‐12, Hamamatsu Photonics) was adopted to measure the forward‐viewing electrical characteristics of those OLEDs at room temperature under ambient laboratory conditions. The active area of the OLEDs is 3 × 3 mm^2^. And four repetitions were carried out for each device and errors in EQE_max_s of those devices were within 5%.

## Conflict of Interest

The authors declare no conflict of interest.

## Supporting information

Supporting Information

## Data Availability

The data that support the findings of this study are available from the corresponding author upon reasonable request.
