# Supplementary material for: Highly Efficient and Stable Blue Organic Light‐Emitting Diodes based on Thermally Activated Delayed Fluorophor with Donor‐Void‐Acceptor Motif
Source: Adv Sci (Weinh). 2022 Feb 27;9(12):2106018. doi: 10.1002/advs.202106018 (PMC9036013; doi:10.1002/advs.202106018)
Supplement: Supplementary file 1 — Supporting Information [file ADVS-9-2106018-s001.pdf]

Supporting Information

Highly efficient and stable blue organic light-emitting diodes based on thermally activated delayed fluorophor with donor-void-acceptor motif

Dongdong Zhang, Yoshimasa Wada, Qi Wang, Hengyi Dai, Tianjiao Fan, Guoyun Meng, Jinbei Wei, Yuewei Zhang, Katsuaki Suzuki, Guomeng Li, Lian Duan, Hironori Kaji*

Synthesis of 2,6-bis(3,6-diphenyl-9*H*-carbazol-9-yl)-9*H*-xanthen-9-one (23PCX):

Under the protection of Ar atmosphere, with stirring, a solution of palladium(II) acetate (20.2 mg, 0.09 mmol) and of tri-*tert*-butylphosphine (66.8 mg, 0.33 mmol) in 20 mL of toluene was added to a solution of 2,6-dibromo-9H-xanthen-9-one (1.17 g, 3.3 mmol), 3,6-diphenyl-9H-carbazole (2.13 g, 6.6 mmol) and sodium *tert*-butoxide (0.86 g, 9.0 mmol) in 20 mL of toluene under room temperature. Subsequently, the mixture was stirred and refluxed (80 °C) for 24 hours before it was partitioned between chloroform and water. The organic layer was separated, and the aqueous layer was extracted with chloroform. The combined organic layers were washed with brine, dried over Mg_2SO_4, and followed with solvent-removal under reduced pressure. Crude product was finally purified by column chromatography (silica, dichloromethane (DCM)/petroleum ether = 1:2) as an orange solid (2.24 g, 2.7 mmol) with yield of 82%.

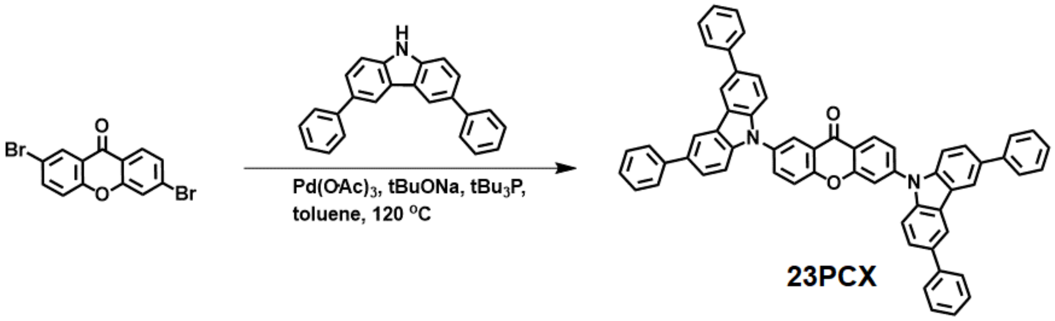

Scheme S1. Synthetic pathway of 23PCX.

23PCX ($C_{61}H_{38}N_2O_2$) ^{1}H NMR (600 MHz, chloroform-D) δ: 8.674-8.645 (m, 2H), 8.427 (s, 4H), 8.044-8.025 (m, 1H), 7.914-7.911 (d, 1H), 7.841-7.820 (d, 1H), 7.802-7.785 (m, 1H), 7.760-7.739 (m, 12H), 7.722-7.707 (m, 2H), 7.537-7.485 (m, 10H), 7.390-7.355 (m, 4H). ESI-TOF (positive mode): calculated as 830.29, found as 831.30 ($M+H^+$). Elemental analysis (calculated, found for **$C_{61}H_{38}N_2O_2$**): C (88.17%, 88.19%), H (4.61%, 4.58%), N (3.37%, 3.38%).

Synthesis of 3,6-bis(3,6-diphenyl-9*H*-carbazol-9-yl)-9*H*-xanthen-9-one (33PCX)

Under the protection of Ar atmosphere, with stirring, a solution of palladium(II) acetate (20.2 mg, 0.09 mmol) and of tri-tert-butylphosphine (66.8 mg, 0.33 mmol) in 20 mL of toluene was added to a solution of 3,6-dibromo-9*H*-xanthen-9-one (1.17 g, 3.3 mmol), 3,6-diphenyl-9*H*-carbazole (2.13 g, 6.6 mmol) and sodium *tert*-butoxide (0.86 g, 9.0 mmol) in 20 mL of toluene under room temperature. Subsequently, the mixture was stirred and refluxed (80 °C) for 24 hours before it was partitioned between chloroform and water. The organic layer was separated, and the aqueous layer was extracted with chloroform. The combined organic layers were washed with brine, dried over Mg_2SO_4, and followed with solvent-removal under reduced pressure. Crude product was finally purified by column chromatography (silica, DCM/petroleum ether = 1:2) as an orange solid (2.33 g, 2.8 mmol) with yield of 85%.

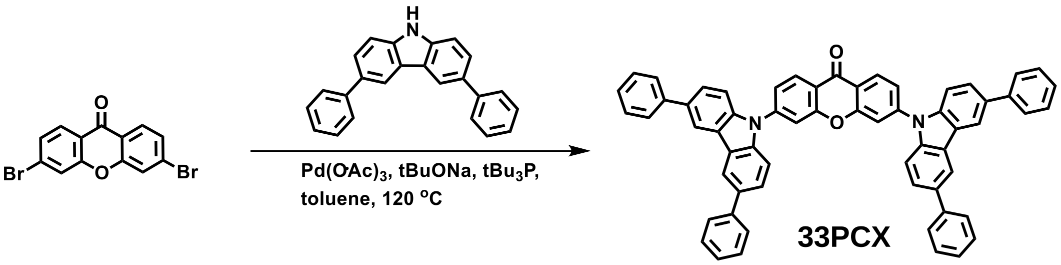

Scheme 2. Synthetic pathway of 33PCX.

23PCX ($C_{61}H_{38}N_2O_2$) ^{1}H NMR (600 MHz, chloroform-D) δ: 8.676-8.661 (d, 2H), 8.418 (s, 4H), 7.874 (s, 2H), 7.801-7.785 (d, 2H), 7.750-7.715 (m, 16H), 7.512-7.486 (t, 8H), 7.386-7.360 (t, 4H). ESI-TOF (positive mode): calculated as 830.29, found as 831.30 ($M+H^+$). Elemental analysis (calculated, found for **$C_{61}H_{38}N_2O_2$**): C (88.17%, 88.18%), H (4.61%, 4.59%), N (3.37%, 3.39%).

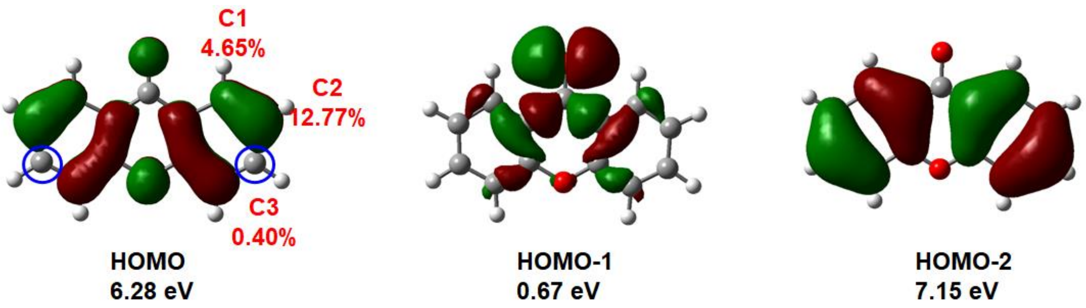

Figure S1. The calculated HOMO, HOMO-1 and HOMO-2 distribution of Xo segment. The distribution of C1 C2 and C3 in HOMO was also provided.

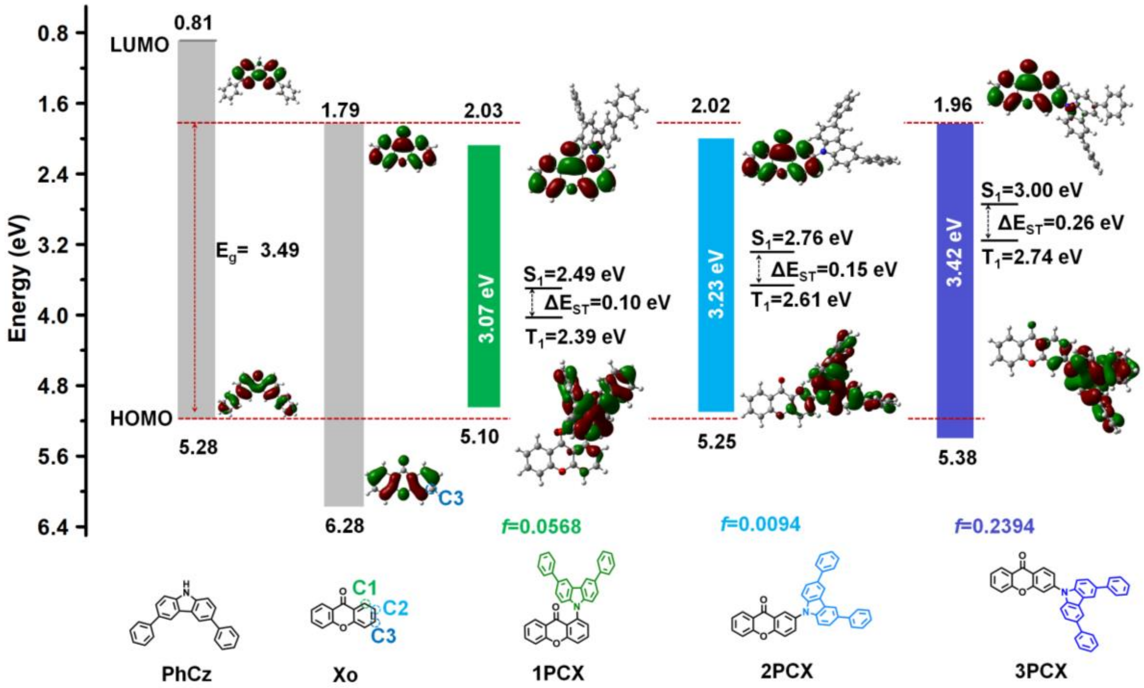

Figure S2. The calculated geometry and electronic properties of 1PCX, 2PCX and 3PCX, respectively.

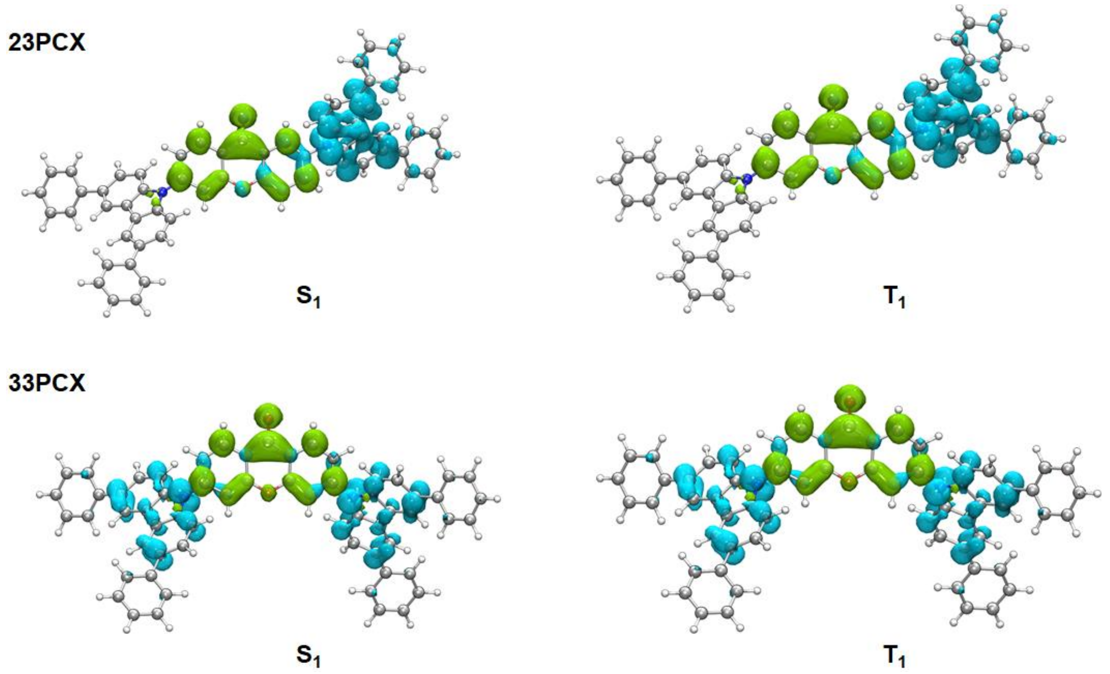

Figure S3. NTO analysis of singlet and triplet of 23PCX and 33PCX.

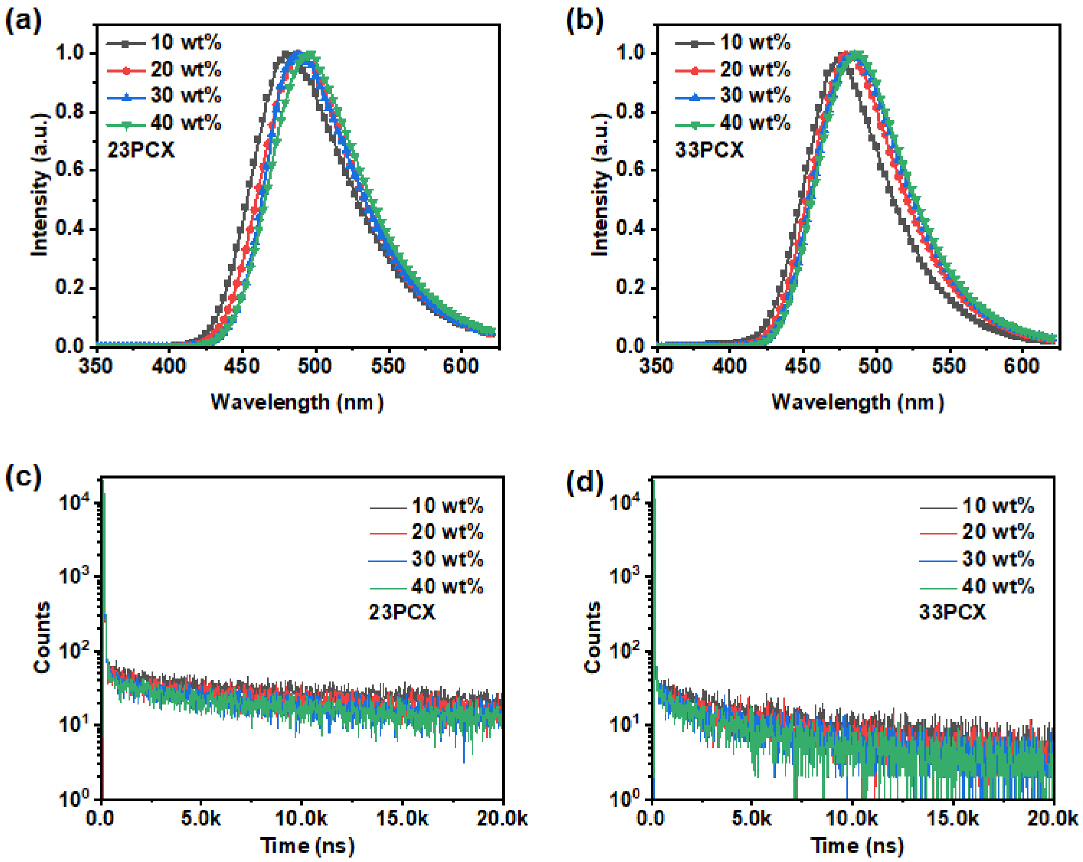

Figure S4. PL spectra of the PPF doped films for (a) 23PCX and (b) 33PCX as well as the PL decay curves of the PPF doped films for (c) 23PCX and (d) 33PCX. The excitation wavelength is 360 nm and the decay curves were recorded at the corresponding emission peaks.

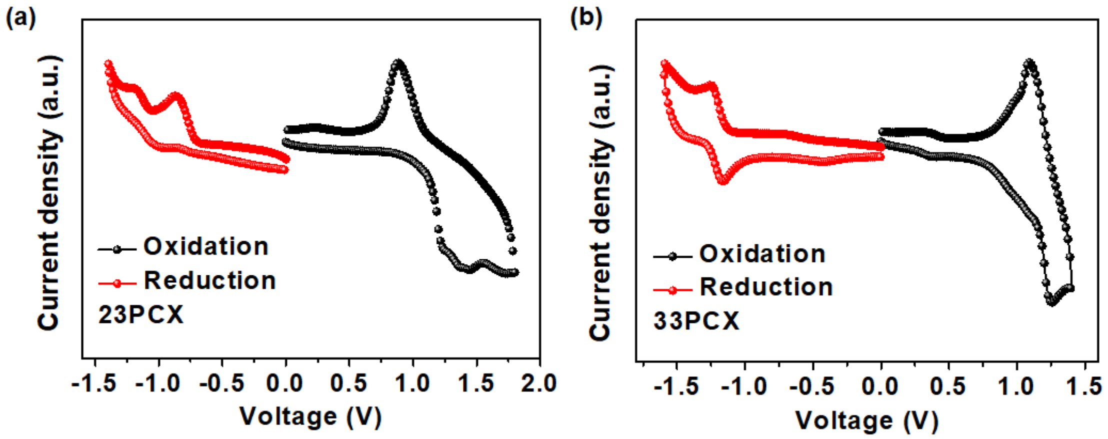

Figure S5. The cyclic voltammetry curves of (a) 23PCX and (b) 33PCX.

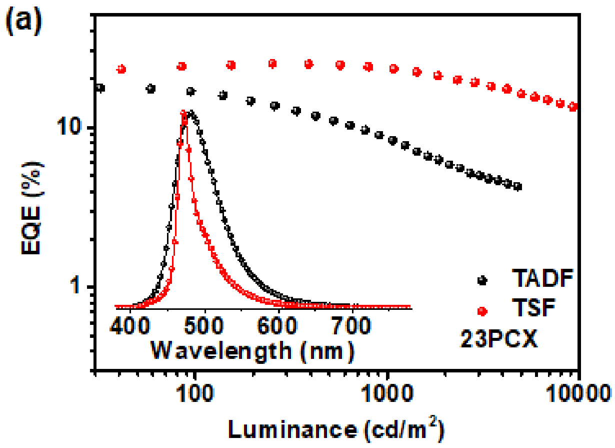

Figure S6. The EQE-luminance curves of devices based on (a) 23PCX and (b) 33PCX as emitter (TADF) and sensitizer (TSF). The inserted figures are the EL spectra (black) of (a) 23PCX and (b) 33PCX, and the EL spectra (red) of ν-DABNA emitted from devices sensitized by (a) 23PCX and (b) 33PCX.

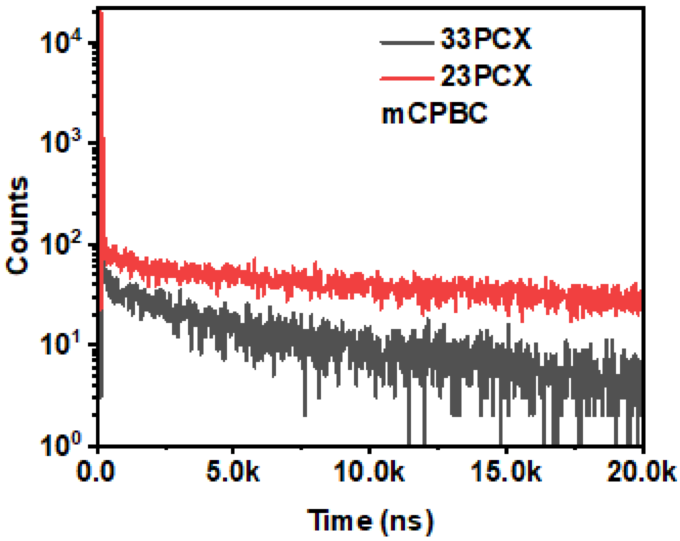

Figure S7. The PL decay curves of mCPBC: 30 wt% 33PCX and mCPBC: 30 wt% 23PCX.

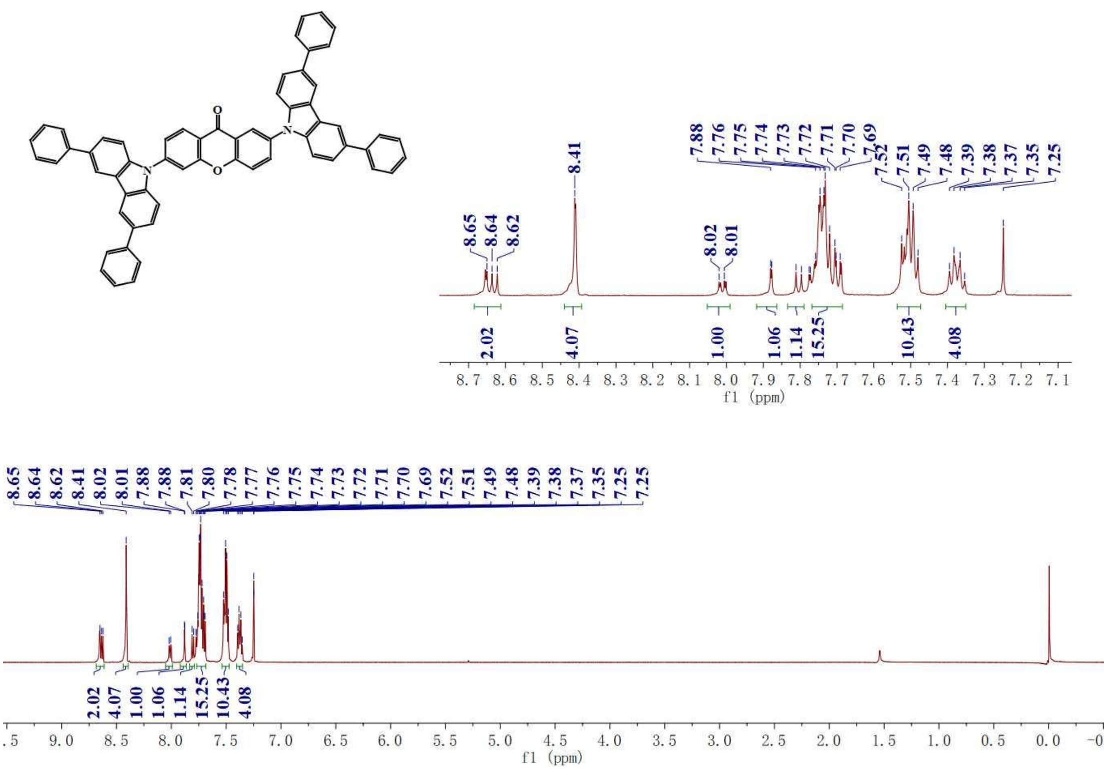

Figure S8. ^{1}H-NMR spectrum of 23PCX.

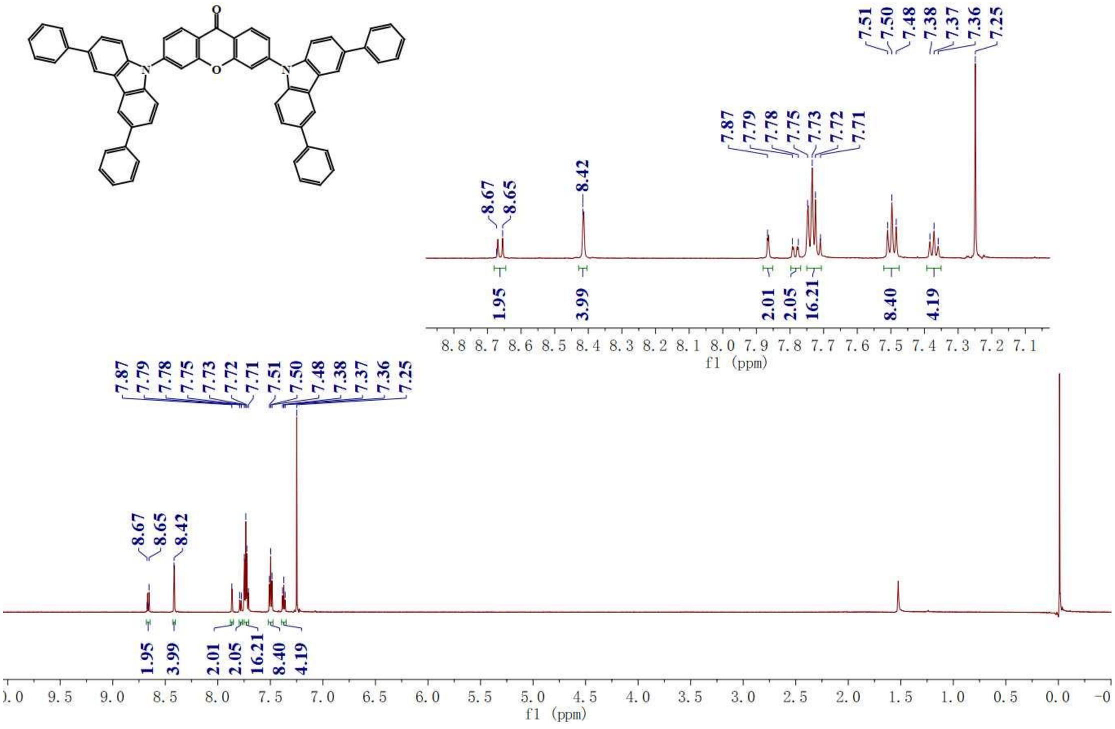

Figure S9. ^{1}H-NMR spectrum of 33PCX.

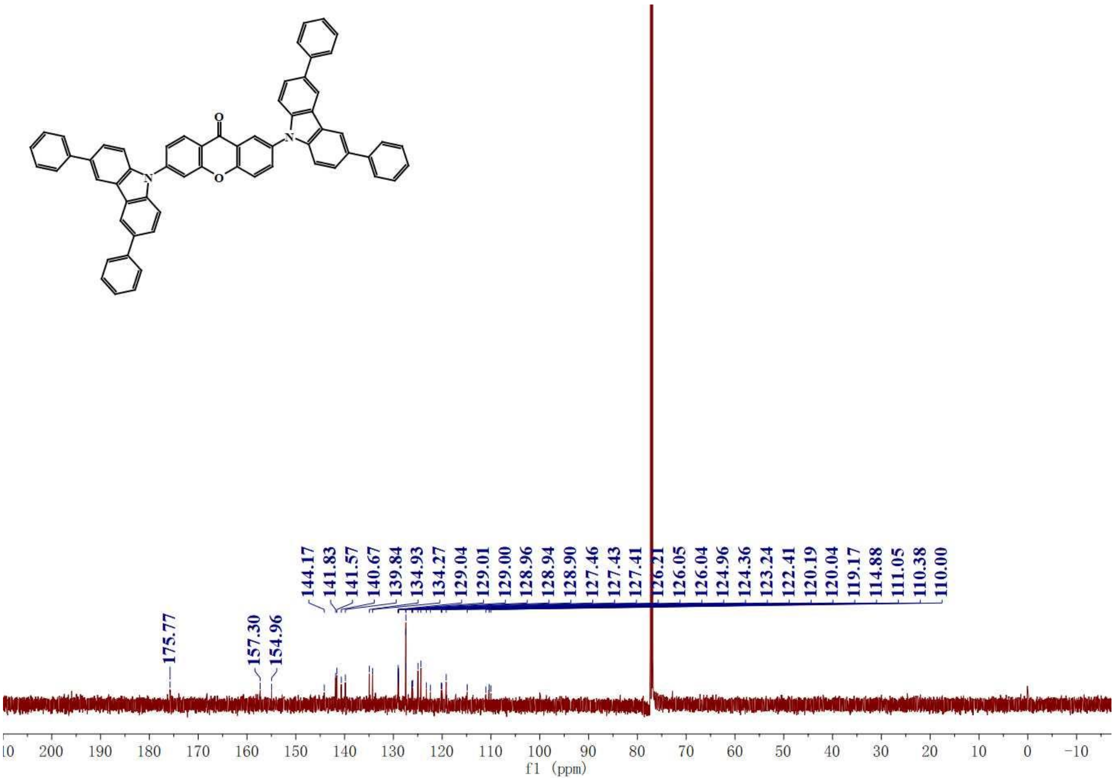

Figure S10. ^{13}C-NMR spectrum of 23PCX.

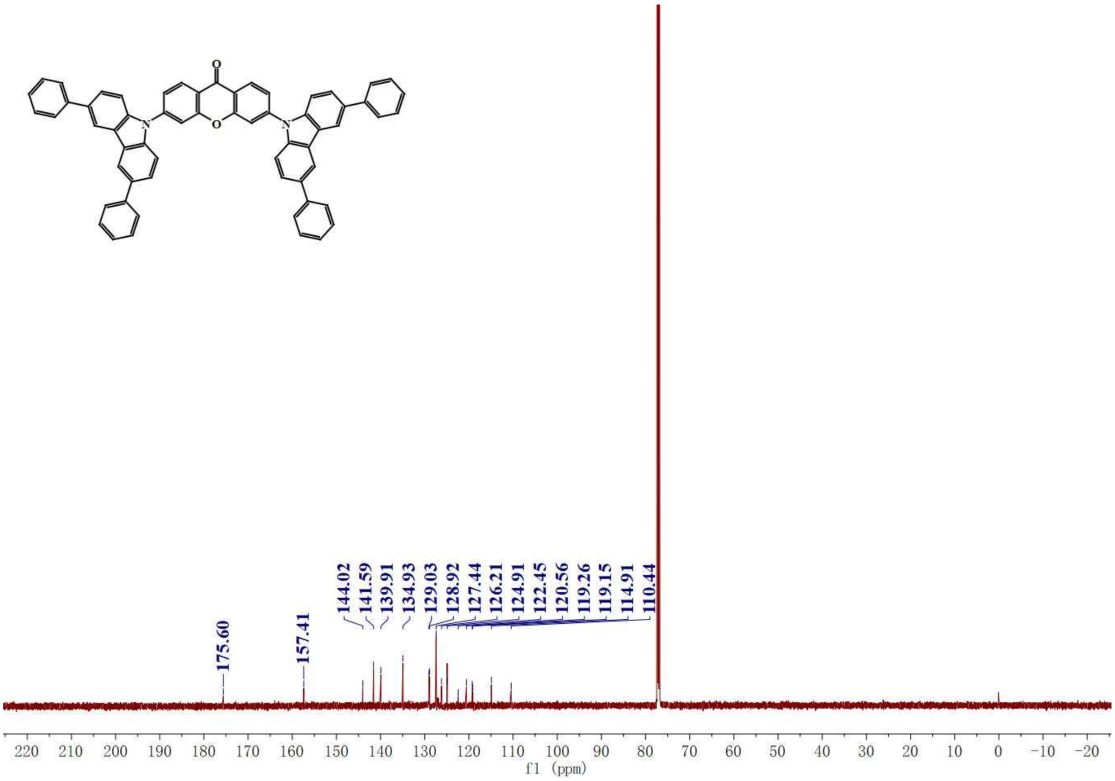

Figure S11. ^{13}C-NMR spectrum of 33PCX.

Table S1: The photophysical properties of doped films.

Film	τ_1 (ns)	τ_2 (μs)	ϕ_{Prompt}	PLQY	k_r (s^{-1})	k_{RISC} (s^{-1})
PPF: 10 wt% 23PCX	17.6	6.78	0.18	0.82	1.0×10^{7}	6.7×10^{5}
PPF: 20 wt% 23PCX	17.9	5.74	0.19	0.88	1.1×10^{7}	8.0×10^{5}
PPF: 30 wt% 23PCX	19.7	4.91	0.23	0.80	1.2×10^{7}	7.0×10^{5}
PPF: 40 wt% 23PCX	19.8	4.90	0.27	0.76	1.4×10^{7}	5.7×10^{5}
PPF: 10 wt% 33PCX	12.9	5.35	0.39	0.84	3.0×10^{7}	4.0×10^{5}
PPF: 20 wt% 33PCX	14.7	5.24	0.41	0.92	2.8×10^{7}	4.3×10^{5}
PPF: 30 wt% 33PCX	15.6	4.49	0.45	0.83	2.9×10^{7}	4.1×10^{5}
PPF: 40 wt% 33PCX	15.4	4.21	0.49	0.80	3.2×10^{7}	3.9×10^{5}
mCP: 30 wt% 23PCX	18.1	6.90	0.21	0.82	1.2×10^{7}	5.7×10^{5}
mCP: 30 wt% 33PCX	12.3	5.75	0.40	0.80	3.2×10^{7}	3.4×10^{5}
